# Assessment of Textural Properties of Puffed Corn Cakes during Storage at Different Relative Humidity

**DOI:** 10.3390/foods11182882

**Published:** 2022-09-16

**Authors:** Virginia Sanchez-Jimenez, Tomas E. Gomez Alvarez-Arenas, Marta Rincón, Jose Benedito, Jose V. Garcia-Perez

**Affiliations:** 1 Department of Food Technology, Universitat Politècnica de València, Camí de Vera, s/n, E46022 Valencia, Spain; 2 Institute for Physical and Information Technologies, CSIC, Serrano 144, E28006 Madrid, Spain; 3 Imasdea Innovaciones y Desarrollos Alimentarios S.L, Pol. Ind. Los Llanos de San Pedro, Ctra. Ávila, E40400 Segovia, Spain

**Keywords:** puffed corn cake, texture, water activity, moisture adsorption, modelling, flexion–compression test

## Abstract

Moisture adsorption is considered a critical factor during production and shelf-life of puffed corn cakes (PCC). This study aims to develop and validate an instrumental method and a mathematical model for the characterization of the textural modifications caused by the moisture adsorption in PCC. For that purpose, PCC were stored at different relative humidities to achieve a wide range of water activities (from 0.1 to 0.8 at 22 ± 1 °C). A flexion–compression test was successfully validated in order to characterize the average textural properties of a PCC batch. A mathematical model considering consecutive elastic and plastic zones satisfactorily fitted (average VAR 99.65% and MRE 3.29%) the average stress–strain profiles of PCC and reported useful textural parameters, such as the deformability modulus (*E*), critical strain (*ε_c_*), and *n* curvature parameter. The structural modifications caused by moisture adsorption led to the reduction in *E* and *n* and the increase in *ε*_c_. Even minor changes on the PCC moisture content involve remarkable modifications of the textural properties, which has to be considered for industry and retail distribution.

## 1. Introduction

Demand for ready-to-eat snacks has been on the rise over the last few decades, mainly due to our fast-paced lifestyle. Changes in societal habits require that the food industry adapt to satisfy these new demands. In this context, the food industry has developed healthy and easy-to-handle snacks [1,2]. Puffed corn cakes (PCC) are an example of a novel product that meets these new requirements, since they are considered a healthy on-the-go snack with a long shelf-life [3]. PCC have a very wide target audience, but they are especially aimed at those people concerned about their diet, as well as a celiac wheat-bread substitute.

PCC are extruded food materials composed of corn grains, water, salt, soy lecithin, and vegetable oils [4,5,6]. Extrusion requires corn grain rehydration (up to 0.14 kg water/kg cake) before mixing with the rest of the ingredients [4,6]. During extrusion, the mixture is compressed for a few seconds between two parallel stainless-steel mould-plates heated to between 260–280 °C [7], reaching pressure values of between 180–200 kg/cm^2^. Next, the mould-plates are quickly separated, producing a pressure drop at high temperature, which causes the flash vaporization of water and the corn grain expansion [6,7]. As a result, a solid foam-like product is obtained, characterized by a solid internal framework formed by air-filled cells [8]. This internal structure provides the characteristic volume of puffed corn cakes and, at the same time, a crunchy and fragile texture [9]. The puffing efficacy (expansion volume) during the extrusion process has been analysed in rice [10], corn [4], okara-rice [11], and black rice puffed cakes [3].

The textural properties of PCC are considered one of its main quality attributes [5]. The consumer expects specific texture sensations in this kind of product, such as crispness and crunchiness [8,12,13]. In this regard, compression and bending instrumental tests have been used for the purposes of assessing the textural properties of puffed cakes [6,7,8,11]. In the case of puffed rice cakes, (i) Laurindo and Peleg [8] proposed a compression test, up to 80% strain at 100 cm/min, and a double-power law expression to describe the stress–strain curves (*n* = 6–10 per batch), while (ii) Lewicki and Jakubczyk [14] selected a 3-point bending test to characterize their fracture (at 1 mm/s, *n* = 7 per batch). The crispness has been characterized by Li et al. [5], placing the cakes (*n* = 5 per batch) on a hollow cylinder of 6.9 cm internal diameter and compressing them using a flat-ended plunger (5.8 cm diameter). This method could be classified as a combined compression–bending test. Laurindo and Peleg [8] manifested that when the compression was accompanied by the brittle fracture of the cell walls, force oscillations are usually recorded causing a random noise and large force fluctuations, which is particularly significant in thin specimens. This leads to a wide experimental variability in the assessment of the textural properties of puffed cakes. The experimental variability could be expected to be of larger magnitude in corn cakes than in rice, due to its larger grain size and more uneven surface. Therefore, previous studies addressing the textural properties of PCC have tested a small number of samples per batch. In this sense, and considering the great variability found in this type of product and the dispersion of the measurements, the determination of the optimal number of samples per batch is critical both to achieve an accurate textural characterization and to validate the methodologies for PCC texture assessment.

Water adsorption represents a major issue in snacks such as PCC, playing a relevant role in their shelf-life [15], which may be also influenced by other storage conditions, such as light, temperature, and packaging material [16]. Water intake may occur from the environment because of the defective or delayed packaging of the product, due to an excessively lengthy storage time, or after opening the package for product consumption. Hence, the increase in moisture content and water activity (a_w_) could result in a plasticizing effect in this foodstuff, promoting changes in the material properties [5,8]. Different studies have reported the effect of the water adsorption on the texture of snacks. Roos et al. [17] studied the effect of water plastification on snack crispness, reporting that physical changes occur during moisture adsorption due to an increment in the mobility and structural reorganization of macromolecules. Thus, Wani and Kumar [1] described the reduction in the stiffness of extruded snacks during moisture adsorption throughout storage and its importance for the products’ shelf-life. Furthermore, Katz and Labuza [18] described the relationship between water activity and the loss of the organoleptic acceptability of crispy products. Li et al. [5] studied the effect of water plasticization on PCC texture; however, the number of samples used for the textural analysis was very limited (*n* = 5 per batch), considering the high intra-batch variability that PCC present.

The modelling of PCC stress–strain curves is essential in order to accurately assess their characteristic mechanical pattern and to compute their main textural parameters. In this regard, only Laurindo and Peleg [8] have addressed the modelling of the mechanical response in puffed rice cakes when subjected to an external stress, which shows the lack of mechanistic understanding when assessing the textural properties of this type of extruded products. For this reason, the objective of this study is to validate a particular textural method for the characterization of the textural modifications in PCC caused by moisture adsorption and to develop a mathematical model to describe their mechanical response and identify relevant textural parameters.

## 2. Materials and Methods

### 2.1. Raw Sample

Commercial puffed corn cakes (PCC) were purchased from a local market (Valencia, Spain). The samples were stored at room temperature (22 ± 1 °C) and kept in their original package (4 PCC per pack) until the analysis.

450 commercial PCC samples were used in the present study in order to select the optimal texture method conditions (texture probe and test speed, Section 2.2.1) and to analyse the optimum batch size (Section 2.2.2). In addition, 270 PCC were used in the moisture adsorption test, in which PCC were stored at differing relative humidities (R.H.) from 10 to 90% (*n* = 30 per each R.H. level) (Table 1).

### 2.2. Instrumental Texture Analysis

The textural properties of PCC were measured using a texture analyser (TA.XT2i, Stable Micro Systems, UK), placed in a temperature controlled room (22 ± 1 °C). The complexity and heterogeneity of the PCC morphology led to determination of a specific method of textural analysis for this product. Thus, preliminary tests were carried out using commercial PCC in two stages: (i) the selection of the texture probe set-up and test speed conditions (Section 2.2.1) and (ii) the selection of the number of samples to be analysed (Section 2.2.2).

#### 2.2.1. Selection of the Texture Probe Set-Up and the Test Speed

**Figure 1:**
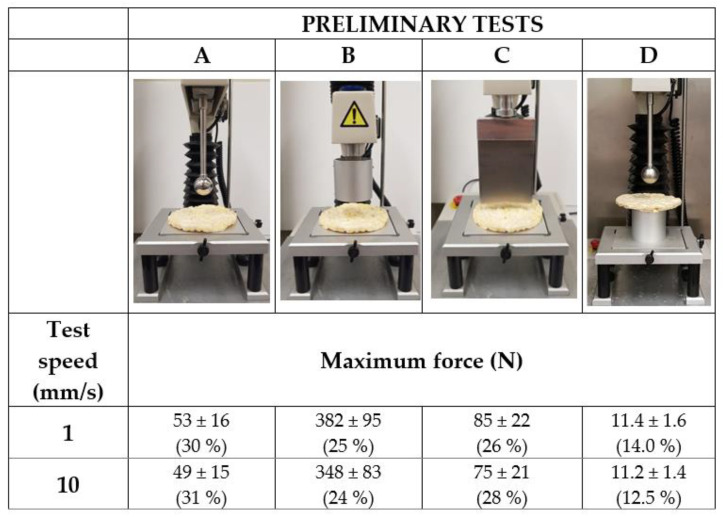
Preliminary tests for texture probe and test speed selection according to the maximum force of compression at two test speeds (1 and 10 mm/s) and 30% strain (*n* = 50). Four texture probe configurations were tested: (**A**) (sphere probe, P/1S), (**B**) (flat cylinder, P/50), (**C**) (Knife blade, A/BS) compressing the cake placed on a flat surface, and (**D**) (Spherical probe, P/1S) compressing the cake placed on a hollow cylinder (50 mm external diameter and 2 mm wall thickness). Mean ± standard deviation and the variation of standard deviation (%) regarding the mean are shown.

Figure 1 shows the four texture probes used in the analysis of snack texture and the two test speeds (1 mm/s and 10 mm/s) chosen for the preliminary tests. Specifically, four different probe configurations were analysed: 2 compression (A and B), 1 shearing (C), and 1 flexion–compression set-up (D). In every case, 50 commercial PCC were tested using a strain of 30%. The A and B compression probes were a spherical probe 25 mm in diameter (P/1S, ANAME, Spain) and a flat-ended probe 50 mm in diameter (P/50, ANAME, Madrid, Spain), respectively, while the shearing probe (C) was a knife blade of 3 mm thickness (A/BS, ANAME, Spain). Those texture probes compressed the puffed cake centre placed over a flat heavy-duty platform (HDP/90, ANAME, Spain). The flexion–compression set-up (D), however, compressed the centre of the sample placed over a hollow cylinder (50 mm external diameter and 2 mm wall thickness) (P/50, ANAME, Spain) with the P/1S spherical probe. The selection of the texture probe and test speed was based on their capacity for a successful coupling between the texture probe and the sample surface and, particularly, on their ability to report the lowest intra-batch variability. In particular, the intra-batch variability was determined by the standard deviation (*sd*) of the maximum force (*F_max_*, N) assessed in the force-time profiles analysed (Exponent Lite^®^, Stable Micro System, Godalming, UK). As will be explained in Section 3.1.1, the D probe at 1 mm/s were the test conditions selected based on these considerations.

#### 2.2.2. Selection of the Batch Size

Once the specific PCC texture method was selected (Figure 1D), the experimental variability remains the main problem to consider in PCC texture analysis. The assessment of the required batch size (*n_s_*, number of samples to be analysed) was then addressed, as it represents an important parameter for the purposes of obtaining representative results. For that purpose, the batch size (*n_s_*) was determined using an inferential statistical method using Statgraphics Centurion XVII (Statgraphics Technologies Inc., The Plains, VA, USA), which is based on Equation (1) [19]. This method consists of estimating a particular parameter of interest from a sample population and, once the confidence level is fixed (95%), its average is compared with that calculated in the batch analysed [20]. In this case, the batch used in Section 2.2.1 (50 PCC) was considered as the population and its average maximum force of compression was compared to that calculated in a small batch (10 PCC).

(1) $$n_{s}={\left(\frac{Z_{\alpha }\cdot sd}{d}\right)}^{2}$$

where *Z_α_* is a standard value (1.96) considering a confidence level of 95% (*α* = 0.05), *sd* is the standard deviation of the batch analysed (10 PCC), and *d* is the precision or the absolute error of the analysis, which is the difference between the maximum compression force for the population (50 PCC) and the batch analysed (10 PCC).

### 2.3. Modelling of Stress–Strain Curves of PCC after Water Adsorption

To our knowledge, no previous literature has thoroughly analysed the mechanical properties of PCC. Laurindo and Peleg [8] proposed a sigmoidal model to mathematically describe the S-shaped stress–strain curves (80% strain) for rice cakes with low and high moisture contents (85% relative humidity). Similarly, other authors [21,22] have modelled the textural properties of expanded snacks based on the statistical Fermi’s distribution function. Thus, in order to better explain the behaviour of PCC when subjected to the validated flexion–compression test (Section 2.2.1 and Section 2.2.2), a mathematical model has been proposed to compute their mechanical properties, and how they are affected by the moisture adsorption. For the purpose of comparison with other materials and previous studies, the stress–strain profile was computed from force-time curves using Equation (2), which represents a modified form of the one used for a 3-point bending test [14].

(2) $$\sigma =\frac{3\cdot F\cdot g}{2\cdot w\cdot L^{2}}$$

where *σ* is the flexion stress (Pa), *F* is the force (N), *g* is the gap of the hollow cylinder (0.05 m), and *w* is the half of the contact distance between the hollow cylinder and the sample ($\pi \frac{D}{2}$) (*D* is the average radius of the cakes 0.1 m) and *L* the cake height (m).

**Figure 2:**
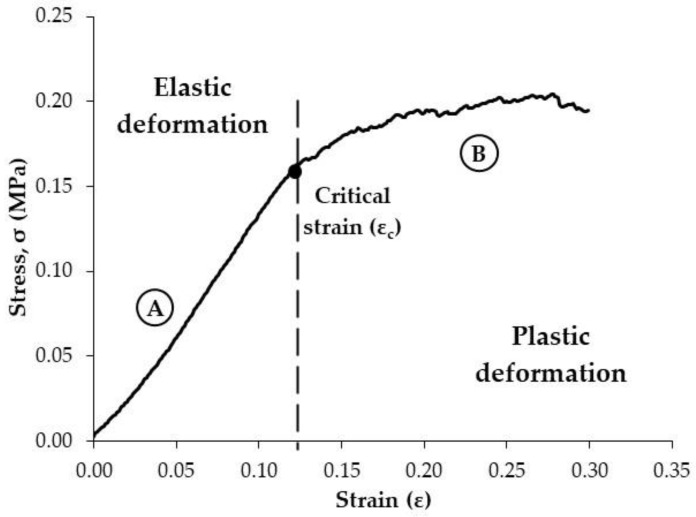
Averaged (*n* = 30) stress–strain profile of commercial puffed corn cakes (PCC) when subjected to the flexion–compression test. Two zones are differentiated: (A) (elastic deformation) and (B) (plastic deformation) bounded when the critical strain is reached.

Specifically, the stress–strain curves obtained were divided into two well-differentiated regions, which refer to elastic (Figure 2A) and plastic (Figure 2B) deformations. On the one hand, the first zone encompasses the elastic region, and it was assumed that the stress increases linearly with the strain and the slope would correspond to the deformability modulus (*E*) [23]. Within the elastic region, no irreversible deformation will take place as the material is elongated [24]. It has to be mentioned that a lag phase is also observed in the curve at very low strains (<3%), corresponding to the coupling of the probe to the surface of the cake. However, it is noted that the spherical shape of the probe facilitates the coupling between the probe and the uneven PCC surface, in contrast with a flat-ended texture probe (Figure 1B). On the other hand, as the deformation of the product progresses (irreversible deformation), random and small fractures lead to a progressive decrease in the apparent deformability modulus, which results in a curvature of the profile (Figure 2B), similar to that found in plastic materials [23].

The stress–strain curves were modelled using Equations (3) and (4). Thus, the characteristic equation of linear elastic behaviour (Equation (3)) was used for describing zone A, while a correction factor in the equation was considered for describing the plastic deformation (concave curvature, zone B) (Equation (4)). Thereby, the *n* parameter is proposed to measure the change of the *E* modulus due to the rate of fracture; thus, the *n* parameter denotes the magnitude of concave curvature in the plastic zone (Equation (4)) (Figure 2). Meanwhile, the separation between the elastic and plastic regions is determined by a yield point (Figure 2), which was defined as the critical strain (*ε_c_*) (Equation (4)).

(3) $$\sigma =E\cdot \varepsilon \;if\;\varepsilon \leq \varepsilon_{c}\;$$

(4) $$\sigma =E\cdot \varepsilon -E\cdot n{\left(\varepsilon -\varepsilon_{c}\right)}^{2}\;if\;\varepsilon >\varepsilon_{c}$$

where *σ* is the stress (MPa), *ε* is the strain, *E* is the empirical deformability modulus (MPa), *ε_c_* is the critical strain, and *n* modulates the magnitude of the concave curvature in the plastic zone.

The parameters obtained after the textural characterization of the PCC and the stress–strain curve modelling were the deformability modulus, the *n* parameter, and the critical strain. The estimation of those variables was carried out by minimizing the sum of the squared differences between the experimental and calculated stresses using the generalized reduced gradient (GRG) method (Microsoft Excel 2019) [25,26]. In order to assess the goodness of the model fit to the experimental data, the explained variance (*VAR*, Equation (5)), and the mean relative error (*MRE*, Equation (6)) were computed.

(5) $$VAR=\left(\frac{1-S_{xy}^{2}}{S_{y}^{2}}\right)\cdot 100\;$$

(6) $$MRE=\frac{\sum_{i=1}^{N}\left|\frac{\sigma_{ex}-\sigma_{c}}{\sigma_{ex}}\right|}{N}\cdot 100$$

where *S_xy_* is the standard deviation of the estimation (MPa), *S_y_* is the standard deviation of the sample (MPa), *σ_ex_* is the experimental stress (MPa), *σ_c_* is the calculated stress (MPa), and *N* is the number of experimental data.

### 2.4. Moisture Adsorption

#### 2.4.1. Adsorption Experiments

For the adsorption experiments, corn cakes were stored under different relative humidity conditions (R.H., %) and at a controlled temperature (22 ± 1 °C) [27]. Saturated salt solutions were used to keep the R.H. conditions constant (LiCl, 10%; CH_3_COOK, 20%; MgCl_2_, 30%; K_2_CO_3_, 40%; NaNO_2_, 60%; NaCl, 70%; KCl, 80%; KNO_3_, 90%) [28]. Salt solutions were located at the bottom of hermetic glass containers and the cakes were placed over a sample stainless-steel holder. Corn cakes were weighed every two days during storage until reaching equilibrium; the storage was extended for up to 15 days for the highest R.H.

Adsorption experiments were carried out in triplicate, testing 10 PCC in each replicate (total of 30 samples for each R.H.). Once the cakes reached equilibrium, instrumental texture tests were performed, and finally, moisture content and water activity were measured.

#### 2.4.2. Moisture Content and Water Activity Measurement

Water activity and moisture content were measured in all of the 10 cakes in each batch after the instrumental texture test. For the measurement of the moisture content and water activity, the cakes were firstly homogenized with a houseware electrical grinder (Blixer 2, Robot Coupe^®^, Vincennes, France). The moisture content was measured following AOAC method 934.06 [29], which consists of drying the cakes in a convection oven at 105 °C until constant weight (24 h), whereas the analysis of the water activity of the cakes was carried out at 22 °C by a dew-point hygrometer (Sprint th 500, Novasina AG, Lachen, Switzerland). Both water activity and moisture content were determined in triplicate.

#### 2.4.3. Modelling the Moisture Adsorption Isotherm

The relationship between the moisture content and water activity at a constant temperature represents the equilibrium isotherm. The adsorption moisture isotherm is mathematically described using the GAB model (Equation (7)) [25,30].

(7) $$W_{e}=\frac{W_{m}CKa_{w}}{\left(1-Ka_{w})(1-Ka_{w}+CKa_{w}\right)}$$

where *W_e_* is the equilibrium moisture content (kg water/kg dry matter), *W_m_* is the monolayer moisture content (kg water/kg dry matter), *a_w_* is the water activity, while *C* and *K* are the parameters related to monolayer and multilayer sorption, respectively. *W_m_*, *C*, and *K* were estimated following the same procedure already described for fitting the stress–strain curve model (Section 2.3).

### 2.5. Statistical Analysis

An analysis of variance (ANOVA) was used in order to determine whether the mean values of the textural parameters identified (the deformability modulus, *n* parameter, and critical strain) were significantly affected by moisture and water activity. A comparison of the means was performed using the Fisher’s Least Significant Difference (LSD) test with 95% confidence interval. A statistical analysis was carried out using Statgraphics Centurion XVII (Statgraphics Technologies Inc., The Plains, VA, USA).

## 3. Results and Discussion

### 3.1. Selection of the Texture Method

#### 3.1.1. Effect of the Texture Probe Configuration and Test Speed

Texture probe and test speed selection for the textural characterization of PCC were based on the sample intra-batch variability. Figure 1 shows the mean and the standard deviation of the maximum force of compression registered for the different probe configurations tested (A–D) at 1 and 10 mm/s for the textural characterization of a batch of 50 PCC. Thus, set-up D presented the lowest standard deviation (14% and 12.5% of the mean value, at 1 and 10 mm/s, respectively). Probes A and B achieved a standard deviation of 30–31% and 25–24% at 1–10 mm/s test speeds, respectively. However, in probe C, the standard deviation was slightly higher at 10 (28%) than at 1 mm/s (26%). This reflects that the test speed had an almost negligible influence on the experimental variability. Thus, the lowest speed condition was selected, according to the textural conditions applied by other authors from previous analyses of this aspect. Orts et al. [6] analysed the texture of puffed rice cakes at 0.083 mm/s and 0.017 mm/s by means of a flexion and a compression test, respectively. Similar conditions were used by Li et al. [5] in the characterization of glassy corn cakes using a flat-ended plunger at 1.7 mm/s. Moreover, the chewing rate for humans is close to 1.4 chews per second in the case of snack products [31], which also reinforces the selection of 1 mm/s for the test speed.

When comparing the different texture probes, a greater variability may be noted in the cases of the compression (probes A and B) and the shearing texture set-ups (probe C) than for the flexion–compression (probe D). The higher accuracy of the measurements when using the spherical probe (P/1S) would probably be linked to its better coupling to the uneven surface of the PCC compared to the other probes. In addition, if configurations A and D are compared, the location of the cake on the hollow cylinder allows for the PCC to respond as a whole during flexion–compression, while in the case of set-up A, the response is more local. This explains the lower degree of variability in configuration D than in A. Based on the aforementioned aspects, the probe configuration D at 1 mm/s was selected.

#### 3.1.2. Influence of the Number of Samples on Texture Analysis

**Figure 3:**
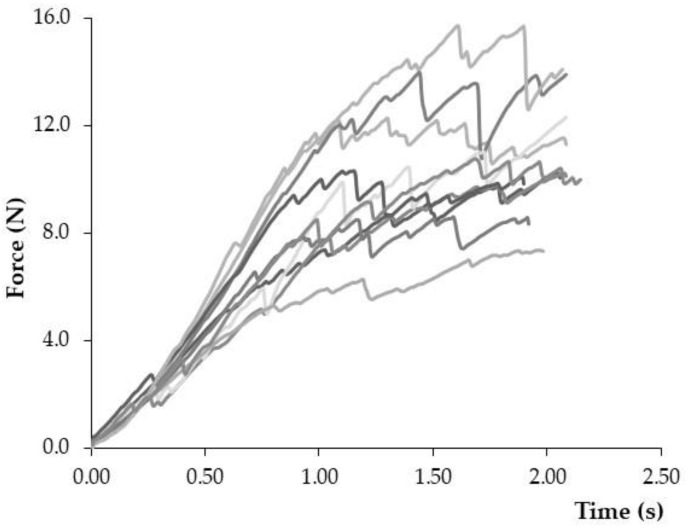
Force-time profile of 10 commercial puffed corn cakes (PCC) obtained by a flexion–compression test (D probe, 1 mm/s up to 30% strain).

The number of samples needed to obtain an accurate and characteristic textural profile is specific for each extruded product. In this regard, Figure 3 shows the force-time profile of 10 PCC from the same batch using probe D. Despite the better performance of probe D’s set-up compared to the others tested (Section 3.1.2), the high intra-batch variability makes it difficult to gain insight into the characteristic mechanical response of the PCC. In this sense, Ulbricht et al. [32] illustrated the critical importance of averaging a representative number of samples to minimize the effect of the random micro-fractures and better understand the batch behaviour in extruded cereal products. Thus, the average stress–strain profile was calculated to better describe the mechanical PCC properties.

According to Equation (1), the batch size (*n_s_*) for a confidence level of 95% was determined by comparing the maximum compression force in a population (50 PCC) to the force computed in a randomly chosen small batch (10 PCC). Thus, from the difference between the average compression forces for the population of 50 PCC (11.4 N) and the small batch of 10 PCC (10.7 N), the *d* value was 0.734 N. Finally, considering the standard deviation of the small batch (2.12 N), the *n_s_* necessary for an accurate textural analysis of PCC was 30.

**Figure 4:**
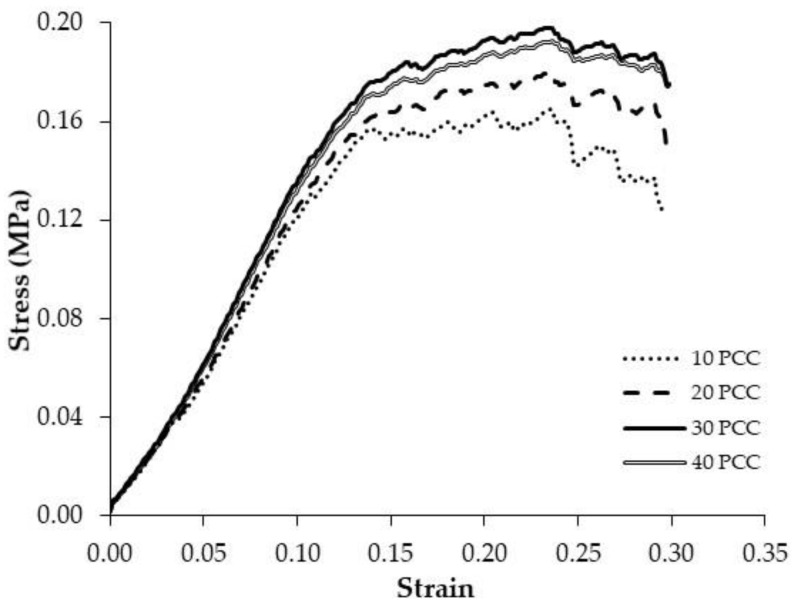
Averaged stress–strain curves of commercial puffed corn cakes (PCC) obtained with the texture method validated (D probe, 1 mm/s up to 30% strain) and using different batch sizes: 10, 20, 30, and 40 PCC.

Figure 4 illustrates the influence that the batch sizes analysed (10, 20, 30 and 40 samples) have on the averaged stress–strain textural profile from a commercial PCC batch. The averaged stress–strain curve, with 10 and 20 samples, presents an irregular profile owing to the highly variable nature of the extruded snack’s structure as a result of the micro-fractures taking place during the flexion–compression test. However, when the number of samples increases to 40 PCC, the shape of the curve becomes smoother and very slight differences were found compared to the curve when using 30 PCC. This supports the selection of a batch size of 30 PCC from the d-value test. In this sense, Heidenreich et al. [33] studied the texture of extruded rice crisps by averaging the textural profile of 10 samples. Laurindo and Peleg [8] analysed the textural profile of puffed rice cakes, using only 6 samples per batch. However, considering the greater structural variability of PCC compared to that of rice, the selection of larger batches seems reasonable [22]. Moreover, it has to be mentioned that Heidenreich et al. [33] and Laurindo and Peleg [8] did not analyse the number of samples to be averaged, which is a necessary step in order to obtain consistent characteristic results.

### 3.2. Adsorption Isotherm

**Figure 5:**
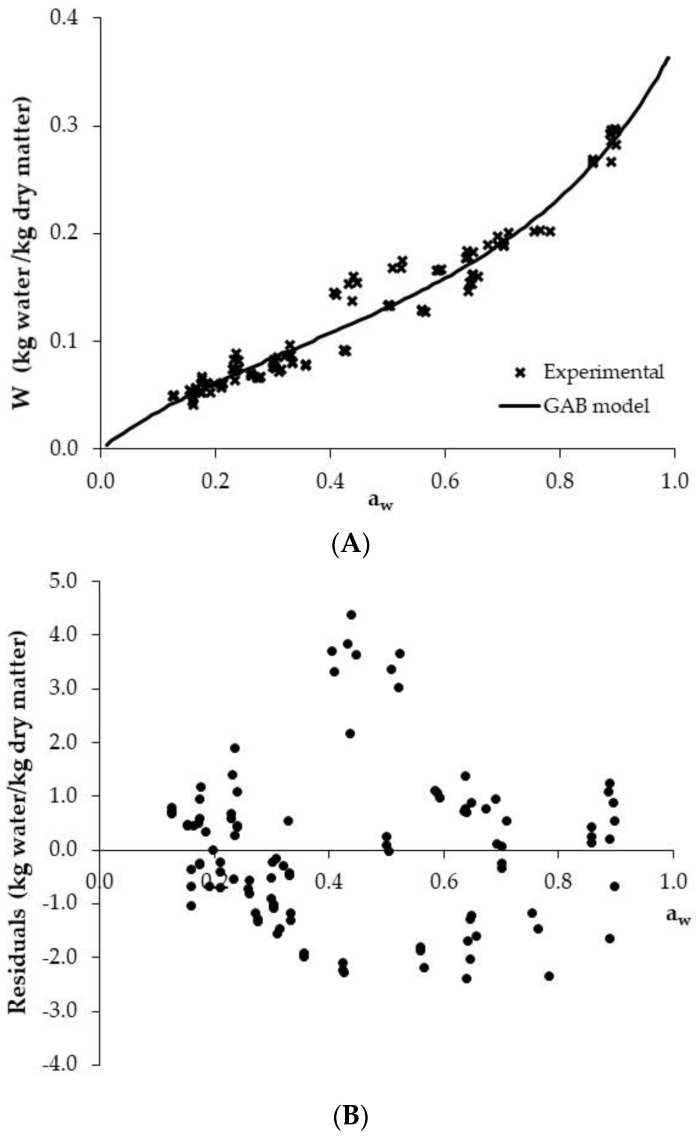
Experimental puffed corn cake (PCC) adsorption isotherm and GAB model, (**A**) and distribution of residuals (**B**).

Commercial PCC presented a moisture content and a_w_ average values of 0.037 ± 0.006 kg water/kg dry matter and 0.126 ± 0.009, respectively (Table 1). Thereby, the samples experimented water adsorption of between 10 and 90% during their storage under controlled conditions of relative humidity. Corresponding points of equilibrium moisture content and a_w_ (22 ± 1 °C) are shown in Figure 5A. PCC adsorption isotherms show a type II sigmoid-shaped curve, characteristic of carbohydrate-rich products, such as extruded snacks [1,34]. Similar S-shaped curves were also observed by Roos et al. [17] and Wani and Kumar [1] when other extruded snacks were analysed.

In the low a_w_ range (from 0.155 to 0.341), small variations in the moisture (from 0.051 to 0.083 kg water/kg dry matter) implied large modifications in the a_w_. Meanwhile, as the a_w_ increased from 0.501 to 0.836, the moisture modifications became progressively larger (from 0.14 to 0.26 kg water/kg dry matter) (Table 1). The adsorption isotherm was satisfactorily described by using the GAB model, *VAR* and *MRE* being of 95.46% and 10.59%, respectively, which reflects a satisfactory fit considering the experimental variability found in the isotherm (Figure 5A). In addition, as illustrated in Figure 5B, the residuals presented a clear random pattern. Therefore, the GAB model allowed a satisfactory mathematical description of the adsorption process in terms of the relationship between moisture content and a_w_.

The monolayer moisture content reported by the GAB model (*W_m_*) for the PCC was 0.11 kg water/kg dry matter, which was similar to that obtained in glassy corn cakes by Li et al. [5], with a value of 0.083 kg water/kg dry matter (R^2^ = 0.93). The importance of the W_m_ value in snacks was manifested by Jensen and Risbo [35], who related textural parameters such as crispness, with the *W_m_* value based on the interactions between the carbohydrate matrix and the monolayer water molecules. As for the *C* and *K* parameters computed by the GAB model, they presented values of 5.19 and 0.72, respectively. Those parameters are associated with the energy of the interactions between solid matrix sites and water molecules in the monolayer and multilayer, respectively [30]. Similar values for cereal-extruded snacks were observed by Wani and Kumar [1], with values of 12.47 and 0.78 for the *C* and *K* parameters, respectively. In glassy PCC, Li et al. [5] reported GAB model parameters (*C* = 3.47 and *K* = 0.88) that were very close to the ones obtained in the present study.

### 3.3. Influence of the Moisture Adsorption Process on the Experimental Stress–Strain Curves

**Figure 6:**
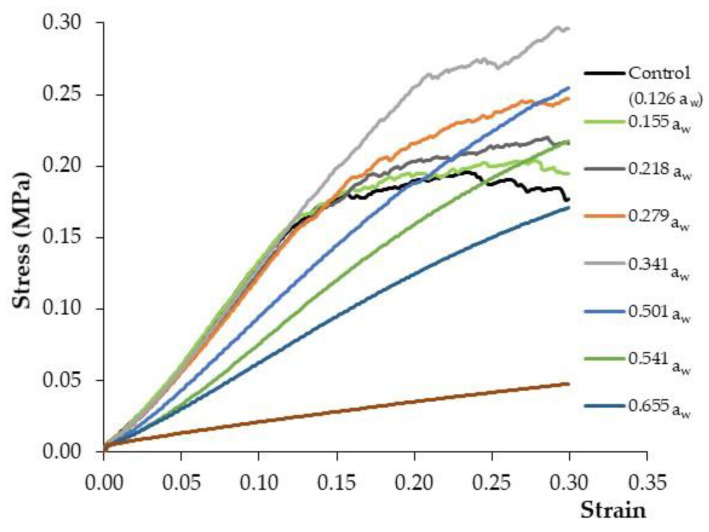
Stress–strain profile of commercial puffed corn cakes (PCC) stored using different salts to reach a wide range of water activity (a_w_ from 0.1 to 0.8) and compared with the control sample (0.126 a_w_).

Figure 6 shows the average (*n* = 30) stress–strain profiles for PCC with different a_w_ values, ranging between 0.1–0.8, and the control PCC (a_w_ 0.126). Firstly, it has to be noted that despite the similarity between the a_w_ value of the control (0.126) and that of the PCC stored at 10% R.H., the slight increase in the moisture content (from 0.037 to 0.051 kg water/kg dry matter) led to a noticeable modification of the stress–strain profile, as can be observed in Figure 6. Secondly, Figure 6 clearly shows two different types of behaviour, one for corn cakes with low a_w_ (below 0.35) and another for PCC with an a_w_ above 0.50. Thereby, for a_w_ < 0.35, the increase in the stress at low strains, following the pattern of elastic materials, was extended until the mechanical stress induced small micro-fractures. This led to a progressive decrease in the deformability modulus, reflected in the transition from a linear to a concave function, following the typical pattern of plastic materials, as explained in Section 3.2 for commercial PCC. The critical strain is related with the yield point of the textural profile that determines the transition of PCC from elastic to plastic material properties. In the plastic zone, as the strain increases, the deformability modulus is progressively reduced and some of the batches almost reach an asymptotic value or plateau with a slope close to 0. This reflects that the breaking point of the cake is near and could be computed by the location of the maximum stress. Thus, an increase in the maximum stress is observed from 0.196 MPa in the control samples to 0.204 MPa and 0.297 MPa in samples with a_w_ values between 0.155 and 0.341, respectively. The observed increase in the maximum stress has been reported by other authors working on similar expanded snacks at low levels of moisture adsorption [23,36,37]. In fact, Barrett and Kaletunc [36] showed that moderate water adsorption in extruded corn snacks does not necessarily produce a softening of the samples, but rather a change in the distribution of fracture intensity. Thus, Mazumder et al. [38] reported an increase in the maximum compression force in corn snacks during water adsorption (from 2 to 10%) from 11 to 39 N, which was attributed to a mechanical transformation from a brittle to a ductile material. A feasible explanation of the increase in the apparent elastic modulus brought about by moisture adsorption is related to a local microscopic softening of the material cell walls, which at macroscopic level induces a greater resistance of the material to the fracture.

At high a_w_ values, from 0.50 to 0.85, the elastic behaviour is more noticeable at higher strains [23] and the plastic zone is displaced to greater strain values in some cases (a_w_ 0.836), beyond the experimental range. In addition, curve smoothing was manifested at high moisture contents, which is a characteristic property of soft materials where microfractures are not easily produced. This is related to the reduction in the frequency of small and random fractures in the PCC during the flexion–compression test. In this sense, Li et al. [5] reported that corn cakes with an a_w_ value of under 0.3 presented irregular jagged force-time curves, whereas the profile of samples above 0.5 a_w_ showed a smooth profile.

### 3.4. Modelling of Stress–Strain Curves and Modification of Textural Properties by Moisture Adsorption

**Figure 7:**
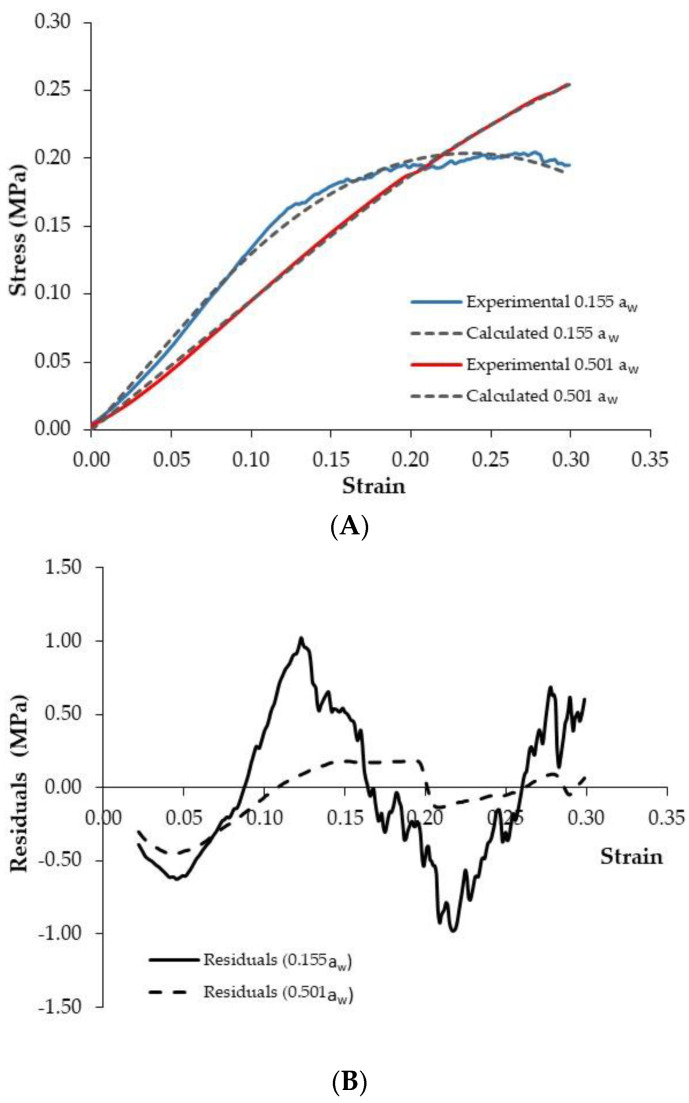
Experimental and calculated stress–strain profile of puffed corn cakes (PCC) (*n* = 30) stored at 0.155 and 0.501 water activity (**A**) and distribution of residuals (**B**).

In order to determine the characteristic mechanical parameters of PCC and how they were modified by moisture content, the mathematical model proposed in Section 2.3 (Equations (3) and (4)) was fitted to the experimental stress–strain curves. Afterwards, the characteristic textural parameters, such as the deformability modulus (*E*), *n* parameter, and critical strain (*ε_c_*), were identified. The proposed model allowed a satisfactory description of the stress–strain data for PCC in a wide range of a_w_ values, with an average explained variance of 99.65% and a *MRE* of 3.29% (Table 1). Figure 7A illustrates the accuracy of the model for both low (0.155 a_w_) and high (0.501 a_w_) water activity batches, while Figure 7B shows the distribution of residuals. Previous mathematical models proposed by other authors [8,22,32] for the purposes of characterizing the textural properties of snacks during moisture adsorption have been validated for small groups of samples or over a small range of a_w_. Furthermore, the model proposed by Ulbricht et al. [32] could not be used for PCC texture modelling because it was developed to estimate the mechanical response of brittle products with a uniform particle size and distribution. For that reason, it was necessary to develop a specific model for PCC texture characterization during moisture adsorption.

**Figure 8:**
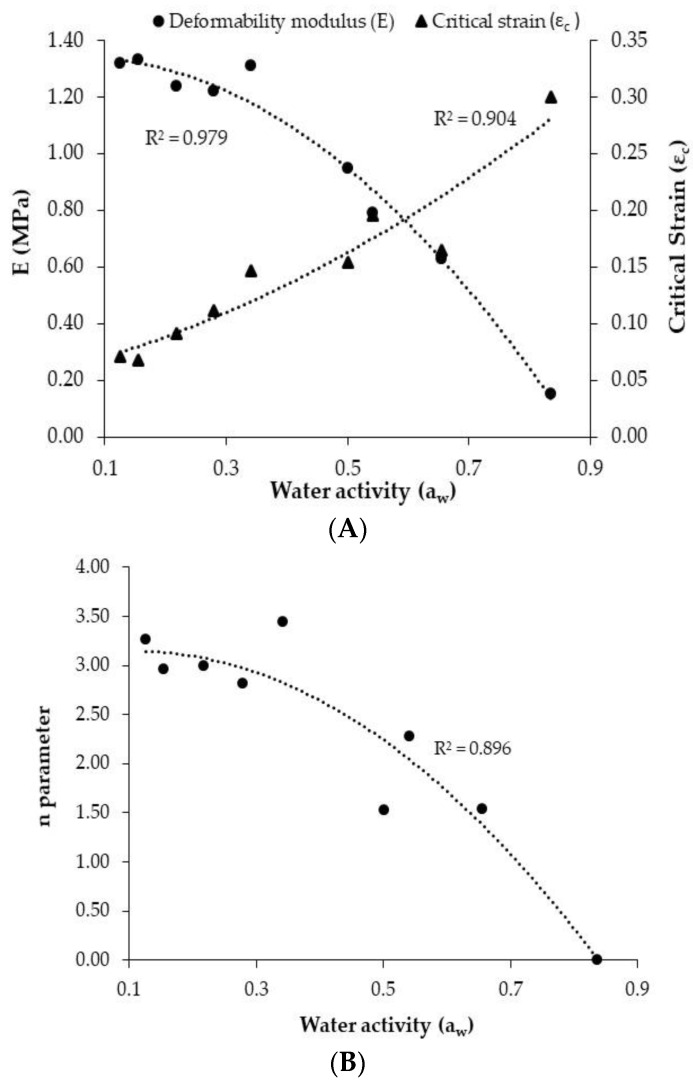
Relationship of the deformability modulus (*E*) (**A**), the critical strain (*ε*_c_) (**A**) and the curvature parameter (*n*) (**B**) with the water activity (a_w_) of puffed corn cakes (PCC).

Table 1 shows the textural parameters obtained for PCC from the proposed model at different water activities. Two distinct types of behaviour can be distinguished for the deformability modulus depending on the a_w_ value. Specifically, samples with low a_w_ (<0.35) presented a fairly constant deformability modulus (*E*), ranging between 1.22 and 1.33 MPa. This reflects that a low moisture adsorption (moisture change from 0.051 to 0.083 kg water/kg dry matter) involves the maintenance of the structural resistance of the cake, considering the *E* of the control sample (1.32 MPa). However, when the R.H. is above 60% during storage, a gradual decrease in the *E* with the water activity was found to take place, with figures ranging from 0.95 MPa (0.501 a_w_) to 0.15 MPa (0.836 a_w_) (Table 1). This behaviour is illustrated in Figure 8A, which shows a satisfactory second-degree polynomial relationship (R^2^ = 0.98) between *E* and water activity. A similar tendency was reported in corn–rye bread by Lewicki and Jakubczyk [14], where moisture adsorption led to a reduction in the elastic modulus from 6 MPa to 1.5 MPa in samples stored at 0.2 and 0.6 R.H., respectively. Saleem et al. [39] studied the effect of moisture on the mechanical properties of sweet biscuits, obtaining E values of between 74 MPa and 5.5 MPa in samples with 0.04 kg water/kg dry matter and 0.1 kg water/kg dry matter, respectively. A reduction in the stiffness of crispy bread during water adsorption was determined by Roudaut et al. [40], quantifying an elastic modulus decrease from 18 MPa in samples with 0.075 kg water/kg dry matter to 5 MPa in samples with over 0.12 kg water/kg dry matter. At the same time, a lower deformability modulus (0.008 MPa) was reported by Laurindo and Peleg [8] in compression tests carried out on puffed rice cakes. However, higher figures of deformability modulus have also been reported for similar products, such as crispy rye bread (95 ± 27 MPa in a bending test) [41]. The differences in magnitude between the figures found could be linked to the different texture probe configuration and the test conditions used.

A similar trend in *n* parameters to that found for *E* is observed when distinguishing two groups of samples according to their water activity. Samples with a water activity of between 0.155 and 0.341 presented a similar *n* value (2.96 and 3.44, respectively). Meanwhile, a gradual decrease in the *n* parameter from 3.26 value in the control sample (0.126 a_w_) to 1.53 (0.501 a_w_), 2.28 (0.541 a_w_) and 1.54 (0.655 a_w_) in samples stored at a R.H. of over 60% is observed. Specifically, the *n* parameter of the samples with a_w_ 0.836 could not be computed since the elastic region was extended up to the end of the deformation, reporting a critical strain of 0.30. Figure 8B shows the relationship between the *n* parameter and the water activity, which was modelled by a second-degree polynomial equation (R^2^ = 0.90). This relationship could be understood by considering that the rate of deformability decay, as a consequence of the random fractures, is of greater magnitude at low moisture contents, at which a greater fragility and crispness of the snack is found. However, the significant decrease in the *n* parameter is evidenced in samples with a_w_ of over 0.35, at which point the PCC presents a higher critical strain and the curvature of the stress–strain profile is reduced.

As regards the critical strain, it presented an upward trend as the a_w_ increased (Table 1). The control commercial PCC presented a critical strain of 0.071, which increased gradually up to 0.30 in the samples with the highest water activity (0.836 a_w_). This evolution was also satisfactorily described by a second-degree polynomial equation (Figure 8A, R^2^ = 0.90). Thus, a shortening of the plastic zone, as the corn cake moisture increased, was clearly manifested in the stress–strain curves (Figure 6). This entails a reduction in the extruded snack’s fragility and a delay in the appearance of the random small fractures, leading to the curvature of the stress–strain curves.

## 4. Conclusions

Puffed corn cake (PCC) texture was satisfactorily characterized by a specific flexion–compression instrumental test, which provided the lowest experimental variability compared to other instrumental configurations. Commercial PCC presented a large experimental variability as a consequence of the uneven surface and the random small fractures that appear in samples with low water activity. Consequently, it is necessary to average a large batch (30 PCC) in order to accurately describe their mechanical behaviour. The mathematical model proposed, which includes the deformability modulus, the critical strain, and the *n* parameters, demonstrated an accurate description of PCC mechanical modification during moisture adsorption. The proposed model illustrated how moisture adsorption involves a reduction in the deformability modulus and a shortening of the plastic zone. Modifications of textural properties linked to the increase in moisture content have to be considered for industry and retail distribution. Therefore, the methodology used in this study, which takes into account the averaging of the stress–strain curves and the mathematical modelling of the textural behaviour, could be considered a feasible method for quality control purposes during the manufacturing and commercialization of PCC.

## Figures and Tables

**Table 1:** Results for the adsorption experiments in puffed corn cakes and textural model parameters.

Salts	R.H. (%)	a_w_	Moisture (kg Water/kg Dry Matter)	*E* (MPa)	*ε*_c_	*n*	VAR (%)	MRE (%)	L (mm)
Control	-	0.126 ± 0.009 ^a^	0.037 ± 0.006 ^a^	1.32	0.071	3.26	99.2	3.58	7.02 ± 0.76 ^b^
LiCl	10	0.155 ± 0.018 ^a^	0.051 ± 0.005 ^a^	1.33	0.068	2.96	99.1	3.87	7.44 ± 0.65 ^c,d,e^
CH_3_COOK	20	0.218 ± 0.036 ^b^	0.065 ± 0.008 ^b^	1.24	0.091	3.00	99.5	3.68	7.54 ± 0.69 ^d,e^
MgCl_2_	30	0.279 ± 0.047 ^c^	0.076 ± 0.006 ^b,c^	1.22	0.111	2.82	99.8	2.78	7.56 ± 0.48 ^e^
K_2_CO_3_	40	0.341 ± 0.045 ^d^	0.083 ± 0.008 ^c^	1.31	0.147	3.44	99.6	3.90	7.18 ± 0.70 ^b,c^
NaNO_2_	60	0.501 ± 0.067 ^e^	0.142 ± 0.016 ^d^	0.95	0.154	1.53	99.9	2.71	7.52 ± 0.56 ^d,e^
NaCl	70	0.541 ± 0.092 ^e^	0.151 ± 0.013 ^d^	0.79	0.196	2.28	99.8	4.82	7.47 ± 0.46 ^c,d,e^
KCl	80	0.655 ± 0.086 ^f^	0.185 ± 0.012 ^e^	0.63	0.165	1.54	99.9	0.93	7.20 ± 0.73 ^b,c,d^
KNO_3_	90	0.836 ± 0.082 ^g^	0.262 ± 0.039 ^f^	0.15	0.300	-	99.4	3.20	4.76 ± 0.86 ^a^

R.H.: relative humidity; a_w_: water activity; *E*: Deformability modulus; *ε*_c_: Critical strain; *n*: curvature parameter; VAR: Explained variance; MRE: Mean relative error; L: thickness; -: it was not computed since *ε*_c_ was extended until the end of the test. Mean ± standard error; Values with different letters are significantly different in a same column (*p* < 0.05).

## Data Availability

The data presented in this study are available on request from the corresponding author.
